# Analysis of jute-glass fiber reinforced epoxy hybrid composite

**DOI:** 10.1016/j.heliyon.2024.e40924

**Published:** 2024-12-04

**Authors:** Md Mahadi Hasan, Md Ashraful Islam, Tareq Hassan

**Affiliations:** a Department of Industrial and Production Engineering, American International University-Bangladesh, Dhaka 1229, Bangladesh; b Department of Mechanical Engineering, Khulna University of Engineering & Technology, Khulna 9203, Bangladesh

**Keywords:** Natural fiber, Jute-glass composite, Hybrid composite, Mechanical properties, Microstructure

## Abstract

This study investigated a composite material combining epoxy with hybrid jute (J) and glass (G) fibers. A straightforward and effective fabrication method was employed, utilizing five layers with various reinforcement materials. To identify the optimal combination, a comprehensive series of tests were conducted using a range of characterization instruments, including Scanning Electron Microscopy (SEM), Universal Testing Machine (UTM), pendulum impact tester, density measurement, specific gravity evaluation, water absorption, and swelling thickness tests. The composite's physical, mechanical, microstructural, and fracture properties were thoroughly analyzed. The findings revealed that Type C exhibited the highest impact strength of 378 kJ/m^2^, a Young's modulus of 10.567 GPa, and a flexural modulus of 13.872 GPa. Conversely, Type F demonstrated superior performance in terms of minimal water absorption (5.676 %) and swelling thickness (3.1 %). These results suggest that incorporating glass fibers in the outer layers and using woven jute fibers significantly enhanced mechanical properties while reducing water absorption and swelling. However, the inclusion of short jute fibers led to a decrease in mechanical performance. Microstructural analysis supported these findings, indicating a semi-brittle behavior with increased strength at the outer layers containing glass fibers. The fibers displayed greater strength than the matrix, resulting in matrix phase cracking before the fibers themselves. Overall, the fabricated composites show promising potential for various applications, offering a viable alternative to wood, plastic, or metal materials due to their lightweight nature and improved durability.

## 1 Introduction

It is observed that there has been a significant uptake in the research of composite materials in the last several decades. This is due to their attractive material properties, versatile applications, and low-cost fabrication process compared to monolithic materials. Subsequently, monolithic materials have somewhat reached their optimum, leading to the vast majority of the ongoing research in the field of composite materials [1]. A composite material is a substance made up of two or more materials with better properties than individual materials when utilized separately. Unlike metallic alloys, each material in a composite retains its own chemical, mechanical, and physical characteristics. They are mainly classified as fiber-reinforced and particle-reinforced [2].

In composite materials, various types of fibers are used as reinforcement. They can be natural or synthetic. Due to its lightweight and eco-friendly nature, natural fibers are getting ample attention nowadays [2,3]. Natural fibers are not only cheap but also environmentally friendly, wholly or partially biodegradable, and renewable, which can be applied to attain novel and high-performance polymer materials. In addition, such composites exhibit adequate mechanical properties (i.e., tensile properties, fracture toughness, flexural stress-strain behavior, and fracture strength), rendering them more attractive to other composites. Due to renewability and easy availability in nature, these fibers are used as an alternative to synthetic fibers as a reinforcing medium [4,5].

Natural fibers can be found in sources like animals, plants, and minerals. Silk, wool, and hair are the kinds of fiber found from animal sources. Plant fibers are cellulose or lignocellulose, which are also divided into a variety of kinds. Among those, bast fibers are one of the most popular fibers. Examples of these include jute, flax, hemp, pineapple, sugarcane, oil palm, ramie, kenaf, roselle, mesta etc. [6,7]. Jute, however, is the least expensive among all of them and is abundant in nature. Jute production is mainly concentrated in the Ganges River delta. Bangladesh and India together produce 80 % of the world's total jute supply. Countries like Myanmar, Thailand, and Pakistan also produce in smaller quantities. Chemical composition of jute contains α-cellulose (45–63)%, lignin (21–26)%, and pentosans (18–21)% [8]. Due to its ‘composite-like' structure along with strongly aligned long-chain molecules, jute is considered one of the most robust fibers with very little extensibility showing high flexural and torsional rigidities and thus they are used as a reinforcing agent in polymer composites as considered in this study. The key advantages that glass fibers present include higher tensile strength, low manufacturing costs, great chemical stability, and better performance-to-cost ratio. These rendered them a great candidate for polymer reinforcement. Nowadays, glass fibers are used in a wide variety of areas including aerospace, automotive, marine, athletic and leisure goods, as well as construction and civil engineering. They are also used as filters and fibrous blankets for thermal and acoustic insulation without a matrix. Thus, glass fibers were selected to be used as reinforcements in polymer composites with jute in this experiment.

In modern polymer composites, resin plays a significant role. The human use of resins has a very long history, which has been documented in ancient Greece, Rome, and Egypt. Some synthetic resins possess similar properties to natural plant resins; nonetheless, many are quite different [9]. Synthetic resins have several classes. Epoxy resin is one of the most popular ones. Nowadays, there are plenty of applications of epoxy resin, including coatings such as anti-corrosive epoxy, and epoxy-wearing compounds; metal bonding such as aluminum adhesive; usage in electronics/electrical components, high tension electrical insulators, fiber-reinforced plastic materials, concrete crack repair, structural adhesives, and house repair for the wall. It is even utilized to bind gutta percha in some root canal procedures. Hence, Epoxy Resin is a useful chemical that can be applied to various types of polymer composites [10,11].

There has been a lot of research on polymer composites using different types of natural reinforcements, such as animals and plants. Rao et al. [12] investigated polymer composites reinforced by goat hair and banana fiber and obtained better mechanical properties. Likewise, fibers found from plants, e.g., bast, leaf, seed, wood, fruit, and stalk, are getting much attention as reinforcement in polymer composites due to their easy availability, eco-friendly nature, and simple processing [13,14]. Yadav et al. [15] worked with sisal fiber and developed epoxy polymer composites. They kept the slag weight percentage fixed, varied the sisal fiber percentage, and concluded that adding fibers improves the mechanical properties. Jani et al. [16] studied another leaf type of plant fiber called agave, cultivated mainly in China, Thailand, and Netherlands. Kapok, cotton, loofah, and weed are the kinds of natural fibers found in the seeds of plants. Rice, barley, wheat, oat, rye, etc., fibers are found in the stalks of the plants. Cellulose and lignocellulose can also be found in grass-type plants, for example, bamboo, bagasse, corn, and sabai. Corn grass is another source of natural fiber and can be utilized to produce composites.

Nevertheless, the most frequently used natural fibers are the bast fibers which are extracted from jute, flax, ramie, kenaf, mesta, etc. Because of its strong structure, high strength/weight ratio, exceptional acoustical properties, provision to cut to any specified length, and availability all around the world, bast fibers are now enormously attracting researchers in the field of composite materials [17]. Among all the bast fibers, jute shows several prominent features, as mentioned earlier, and is copiously cultivated in tropical regions, particularly in Bangladesh [18,19]. Having more than 60 % cellulose jute fibers gives high strength in structural applications. Gopal et al. [20] studied the mechanical and flexural properties of jute fiber composites using epoxy and coconut shell powder. They showed that the perfect blending of coconut shell powder offered better tensile and flexural strength with improved delamination characteristics. Gopinath et al. [21] investigated the mechanical properties of jute fiber epoxy composites employing polyester and observed the effect of NaOH concentration on properties. It was discovered that composites with lower NaOH concentrations performed better. Lalta et al. [22] used jute fiber and basalt fiber mats (geo-grid) reinforced with epoxy hybrid composite and examined the influence of stacking sequence on physico-mechanical properties. Arun Premnath [23] studied natural fiber/epoxy resin composites reinforced with sisal and jute and reported an increase of strength and hardness. Seldon and Rajesh [24] studied hemp/rice-husk/E-glass fiber cardanol epoxy hybrid composites and observed improved thermal and dimensional stability due to the superior bonding ability of cardanol leading to better fiber encapsulation. Baky et al. [25] fabricated a fiber-metal laminates based on jute and glass and reported improved mechanical properties. Satapathy et al. [26] analyzed the dynamics of a jute composite reinforced with Si-C derived from rice husk. They observed that adding filler elements improves composites' mechanical properties. Despite noticeable ongoing research on jute fibers, very limited work has been reported on the hybrid composite of jute-glass fibers. Sanjay et al. [27] studied jute-glass fibers using LY556 epoxy resin and HY951 hardener, concluding they had better strength than jute fiber and glass fiber.

Nonetheless, previous investigations lack adequate analysis of such hybrid composites, including considering only a few glass jute fiber layers, conducting a limited number of tests, and the non-presence of microstructural examinations. For example, Sanjay et al. [27] investigated jute/e-glass fiber epoxy composites; however, they employed only two types, leading to inadequate combinations of glass and jute fibers. They also didn't perform any micrographical and fracture analysis. Similarly Ruhul et al. [28] studied jute and E-glass fiber reinforced composite, however, cleary lacks to anlyze the effects of different combinations of the layers, discussion is notably limited, and insufficient evaluation of materials properties. Srivastav et al. [29] fabricated jute/glass hybrid reinforced epoxy composites and analyzed their loading rate sensitivity. However, the authors didn't discuss the influences of using different layers, no proper evaluation of mechanical and physical properties of the composites, and lack of discussion of fabrication method employed. Vijay et al. [30] fabricated jute/hemp/epoxy, hemp/flax/epoxy, and jute/hemp/flax/epoxy hybrid composite, and found the latter one shoing maximum hardness and strength. Though they compared several types of hybrid composites, however, they didn't exclusively examine the different combinations of jute-glass fiber reinforced epoxy hybrid composites. They also lack microstructural and fractural analysis. A number of studies have also been reported to examine the hybrid composite of jute and epoxy; however, they have not examined the effects of glass fiber, such as [26,31], and [30]. The gaps mentioned above lead to the necessity of an in-depth analysis of this composite and exploring its potential applicability in commercial uses. Hence, in this study, we examined five different layers of composites such as Type A (J + J + G + J + J), Type B (J + G + G + J), Type C (G + J + J + G), Type D (J + G + J + G), and Type F (G + J + G + J + G) and performed an extensive analysis of their physical, mechanical, fractural, and microstructural characteristics. For obtaining the optimal combination, several tests were conducted, and a large variety of characterization techniques were implemented. All combinations' densities were measured, their specific gravity was evaluated, water absorption capacity was analyzed, and swelling thickness phenomena were examined. A simple hand lay-up fabrication process was adopted involving four consecutive steps: mold preparation, gel coating, lay-up, and curing. The fabricated composites have significant potential applications in various sectors to replace wood, plastic, or metallic materials for lightweight and better durability, such as table and chair tops, insulation over engine covers in buses and trucks, and the base and coverings of passenger compartments of train and launch cabins.

## 2 Materials and methodology

In this study, woven dry fibers are physically inserted in the mold first, and then the resin matrix is applied using a brush. The wet composite is then rolled with hand rollers for improved contact between the reinforcement and the matrix, facilitating uniform resin dispersion and achieving appropriate thickness. Finally, the laminates are allowed to cure in a controlled environment. The raw materials used include jute yarn, woven glass fiber mats, resin, and hardeners. Jute-yarn of thickness of ∼0.8 mm–1.5 mm was collected from Chawkbazar, Dhaka.

**Fig. 1:**
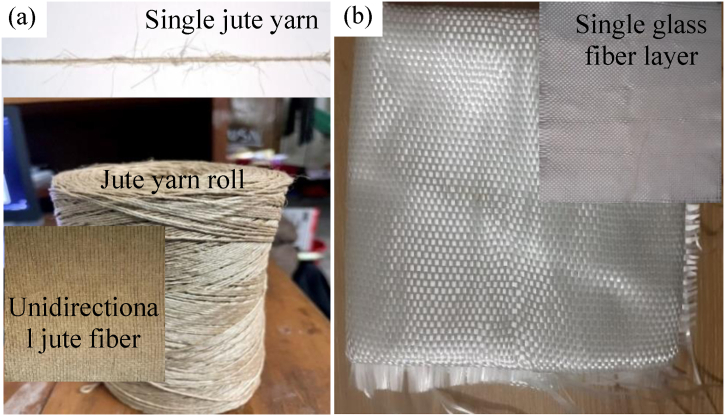
(a) Jute yarn roll and single jute yarn, (b) representative image of 400 GSM glass fiber cloth and single layer.

Commercially available woven bi-directional glass-fiber mats of 400 GSM and 18 GSM were selected as the reinforcement with jute. For matrix materials, epoxy resin with hardener were used in a suitable ratio. Before stacking the plies together, the hardener and resin were thoroughly mixed according to the ratio. Fig. 1(a) shows rolled jute yarn, single jute yarn, and unidirectional jute fiber mate made from jute yarn. Fig. 1(b) presents glass fiber cloth and a cut portion ready for making the composite.

### 2.1 Handloom preparation and making unidirectional jute mats

**Fig. 2:**
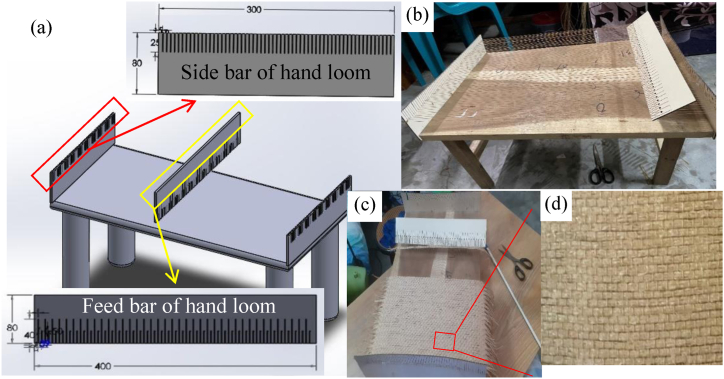
(a) CAD model of simple hand loom showing the teeth of side and feed bar, (b) in-house fabricated simple hand loom setup, (c) making process of unidirectional jute mat, and (d) ready unidirectional jute fiber mat.

The loom is used to weave cloth from yarn. In this experiment, unidirectional jute cloths or mats were needed. For this, a special type of simple handloom was constructed to weave the jute mats from jute yarns. Fig. 2 presents the CAD model and fabricated simple hand loom process. Fig. 2(a) shows the teeth configuration of the sidebar (top) and feed bar (bottom). The sidebars are mounted to a regular table with 5 mm teeth distance from one another and 25 mm deep, as shown in Fig. 2(b). For a straight warp of yarn on the loom, the teeth must be parallel from one side to the other. The jute fibers are woven using a feed bar. The feed bar's up-and-down motion creates space between the warp yarns, allowing the jute fiber to be woven. There are two types of teeth on the feed bar. One type of tooth has a hole to lock the loom's warp yarn during the upward motion of the hand tool. The holes are 1.5 mm in diameter, and the teeth are 20 mm deep, but the other teeth are 40 mm deep and have no holes. The hand tool's teeth are 10 mm apart and are of the same type. The jute mat is woven with jute yarn. Only one unidirectional mat is made at a time using the handloom. Initially, a standard cotton cord was tightened around the teeth of the sidebars from one side to the other. The feed bar was then placed on the chord according to its teeth (Fig. 2(b)). The feed bar was then used to stack 10 jute yarns at a time inside the loop generated by moving it up and down (Fig. 2(c)). Each jute mat was made by repeating this up-down action. Fig. 2(d) presents the magnified image of the unidirectional jute fiber mat. Each mat was kept to the same size (36 × 30 cm). And the thickness was between 1 and 1.5 mm. After weaving, the mat was carefully removed from the loom, and this way, a required number of mats were produced.

### 2.2 Composite fabrication process and layer configurations

**Fig. 3:**
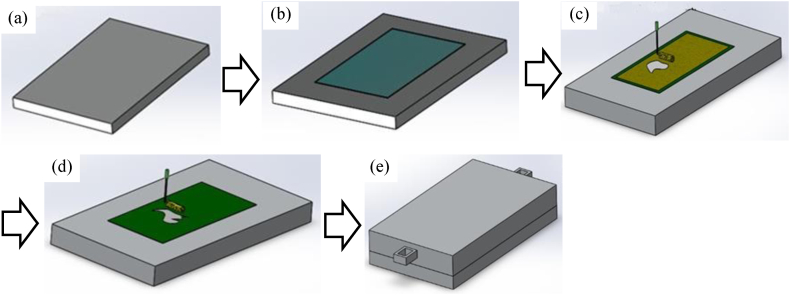
(a) Lower surface plate, (b) polythene over the lower surface, (c) putting jute/fiber glass layer over the polythene according to the orientation and again rolling the mixture over the layer, (d) pouring resin-hardener mixture over the polythene and rolling the mixture, (e) putting the heavy upper surface on the preferred combination of layers.

After preparing the jute mats, they were dried up to remove any trapped moisture. A simple and low-cost hand layup process was used for fabricating the hybrid composite. It is an open molding process, so the composites can be fabricated in any shape; however, for the sake of simplicity, a plate shape was preferred. It begins with a base plate or mold using a polythene cover. Afterwards, 2 kg of LY556 epoxy resin along with 200 gm of HY951 hardener was prepared as matrix materials with a 10:1 resin-hardener ratio and then swirled in a jar for 5–8 min until it became transparent. It is well-known that the mixture is ready for usage when it turns crystal clear and generates heat. To prevent the mixture from setting up inside the jar, it was constantly swirling. Fig. 3(a–e) presents the 3D model of the sequence followed in this experiment. Fig. 3(a) shows the lower surface plate, on which polythene was placed as shown in Fig. 3(b). Then jute and glass fibers were placed as per the orientation (Fig. 3(c)), followed by pouring resin-hardener as mentioned in Fig. 3(d). Finally, the upper surface is place on the top as shown in Fig. 3(e).

In the hand lay-up process, some releasing mixture was put over the polythene sheet so that it didn't stick up with the composites. Then, the piles of fabrics were stacked serially one over the other. Vacuum-assisted Resin Transfer Molding (VaRTM), which impregnates dry fabric with resin, and resin infusion technique, is used broadly for fiber composite fabrications. They are expensive and require a complex setup. In this experiment, a cold press was used to simplify the fabrication process quickly and at a low cost. A heavy flat plate of 60 kg was used as the cold press, which removed the excessive resin-hardener mixture and air voids trapped between the fibers and resin.

**Fig. 4:**
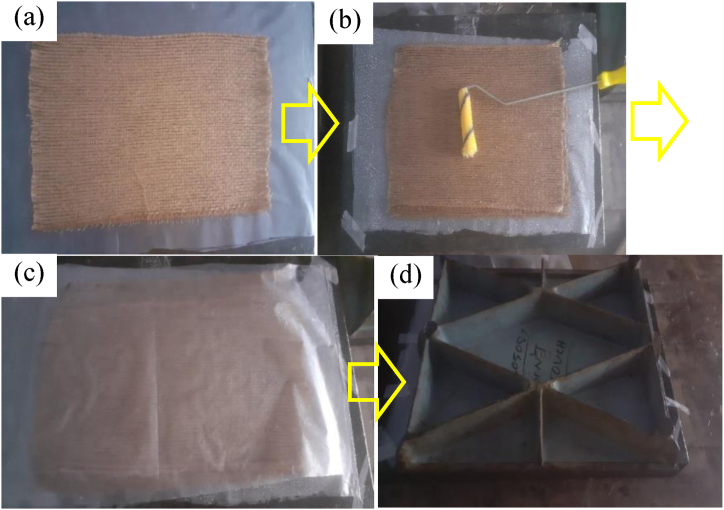
(a) Jute layer over polythene, (b) Rolling resin-hardener mixture with roller, (c) polythene over the top layer, (d) 60 kg weight over the composite.

**Fig. 5:**
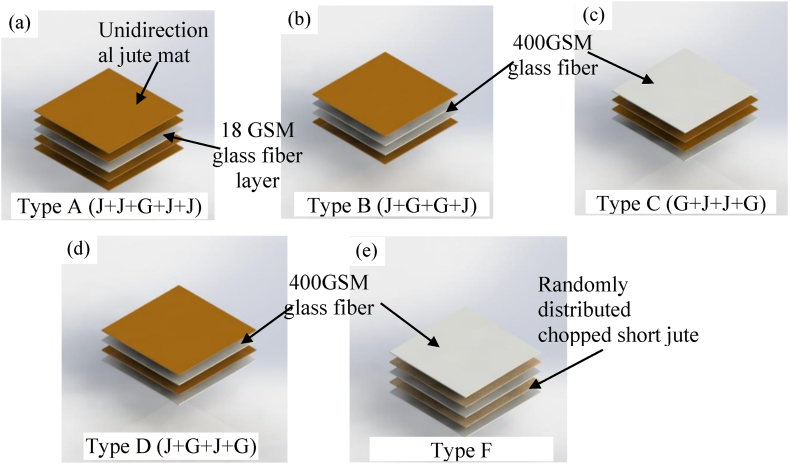
Configuration of fiber piles showing 5 different types of layer combinations.

In Fig. 4, the fabrication process is illustrated. Fig. 4(a) shows the polythene placed on the bottom surface and on which the jute mat is placed. Fig. 4(b) presents orienting the fibers and pouring the resin-hardener mixtures. After putting the preferred combination of fiber layers, another polythene was put on the top as shown in Fig. 4(c). Then, the heavy plate was put on the polythene and kept for 72 h at room temperature so that the resin-hardener mixture set itself up (Fig. 4(d)). After that, the fiber-reinforced epoxy resin composites that were produced were collected and prepared for further processes. Five types of hybrid composites were fabricated. Jute layers were kept in the direction of tensile loading for all the samples, whereas the fiberglass layers were bi-directional. All the layers were kept at the same length and width. Type A is the composition of 4 jute mat layers and 1 layer of 18 GSM glass fiber. Likewise, all the combinations are mentioned in Table 1 with their corresponding thickness, weight fraction (fiber and matrix and density. Fig. 5 shows a 3D representation of the configuration of fiber piles of 5 different layer combinations. Type A, Type B, Type C, Type D, and Type F are shown in Fig. 5(a)–. (b), Fig. 5(c)–. (d), and Fig. 5(e) respectively.

**Table 1:** Arrangement and ingredients of the fabricated composites.

Type of composites	Arrangement of materials	Thickness (mm)	Weight fraction, % (fiber)	Weight fraction, % (matrix)	Density, Kgm^−3^
Type A	J + J + G + J + J	6.63 ± 0.10	29	71	1038.34
Type B	J + G + G + J	4.17 ± 0.08	46.18	53.82	1112.24
Type C	G + J + J + G	3.73 ± 0.10	46.03	53.97	1131.72
Type D	J + G + J + G	4.20 ± 0.07	33.96	66.04	1145.81
Type F	G + J + G + J + G	4.82 ± 0.40	33.33	66.67	1236.34

### 2.3 Physical properties evaluations

Evaluation and justification of the physical properties of a composite material is imperative as it plays a vital role in analyzing the feasibility of using hybrid composites in various practical applications. In this study, all the necessary physical properties tests were carried out, including density evaluation, specific gravity determination, percent of water absorption, and percent of swelling thickness, to facilitate extensive analysis of the fabricated composites. The details are illustrated in the upcoming subsections.

#### 2.3.1 Density measurements

To determine the densities, 3 specimens were prepared from each type of composite. A simple and well-known technique, as shown in Eqn. (1), determines the densities. The volumes of the samples were calculated by measuring the lengths, widths, and thicknesses using a digital slide calliper. Afterwards, their masses were determined by a high precession digital balance. Then, the density of each type of composite was determined by the following formula. The final density was found by taking the average value.

Eqn. 1 $$\rho \left(t\right)=\frac{m}{V}$$

Where, m = mass of each specimen in kg, V = volume of each specimen in m^3^, ρ = density of the composite in kgm^−3^.

#### 2.3.2 Specific gravity determination

Specific gravity is defined as the ratio between an object's density and a reference object's density (water). The dry weight of a specimen was first determined to determine the specific density of each type of hybrid laminate. Then, it was immersed in a 250 ml beaker; its weight was recorded again. Specific gravity was evaluated using the formula mentioned in Eqn. (2).

Eqn. 2 $$S_{p}G=\frac{Dry\;weight}{Water\;weight\;after\;immersion-Water\;weight\;before\;immersion}$$

#### 2.3.3 Water absorption computation

The test samples were immersed in normal surface water and 3.5 % salt water, where the specimens were tested for water absorption examination. At room temperature, five types of composite specimens were immersed in water for 5 weeks. Before the weight measurements, the specimens were taken out of the water and rubbed dry to remove any surface water. The samples were then weighed using an analytical precision scale that was precise to 0.1 mg on a weekly basis. After collecting weekly data, the relative water absorption was calculated using the formula as shown in Eqn. (3). Initial records of each specimen before immersion are presented in Table 2.

Eqn. 3 $$W_{A}\left(t\right)=\frac{W_{n}‐\text{Wo}}{\text{Wo}}*100$$

Where, W_A_(t) = relative water absorption of the specimen after each week, W_o_ = initial specimen weight, W_n_ = specimen weight at each week.

**Table 2:** Masses of the specimens for water absorption analysis.

Specimen types	Mass before immersing in distilled water(gm)	Mass before immersing in 3.5 % salt water(gm)
A	3.66	3.52
B	1.88	2.07
C	2.03	2.05
D	3.47	2.54
F	2.74	3.98

#### 2.3.4 Swelling thickness assessment

As the reinforcement of the fabricated composites is jute, the composites contain cellulose. Cellulose has hydrophilic properties, which means it gets swelled when in contact with water. To evaluate the percent of relative swelling on a weekly basis, the thickness of the specimens was recorded before immersing and weekly after immersion. For evaluating the swelling percentages, the thickness of the specimens was recorded for 5 weeks. The percentage of relative swelling was determined using Eqn. (4). Initial thickness records of each specimen are given in Table 3.

Eqn. 4 $$S_{W}T\left(t\right)=\frac{Tn-T0}{T0\;}*100$$

Where S_W_T(t) = relative thickness of the specimen at each time, T_n_ = specimen thickness after each week, T_o_ = initial specimen thickness.

**Table 3:** The thickness of the specimens for swelling thickness analysis.

Specimen types	Initial thickness before immersing in distilled water(mm)	Initial thickness before immersing in 3.5 % salt water(mm)
A	6.18	6.12
B	4.10	4.12
C	3.50	3.50
D	4.08	4.08
F	4.51	4.56

### 2.4 Mechanical properties evaluation

For determining the tensile and flexural properties of the fabricated composites, ASTM D638 and ASTM D7264 standards were followed, respectively. Additionally, ASTM A370 ASTM D256 was adopted to determine the impact properties. Consequently, specimens were prepared according to the standards.

#### 2.4.1 Tensile properties

**Fig. 6:**
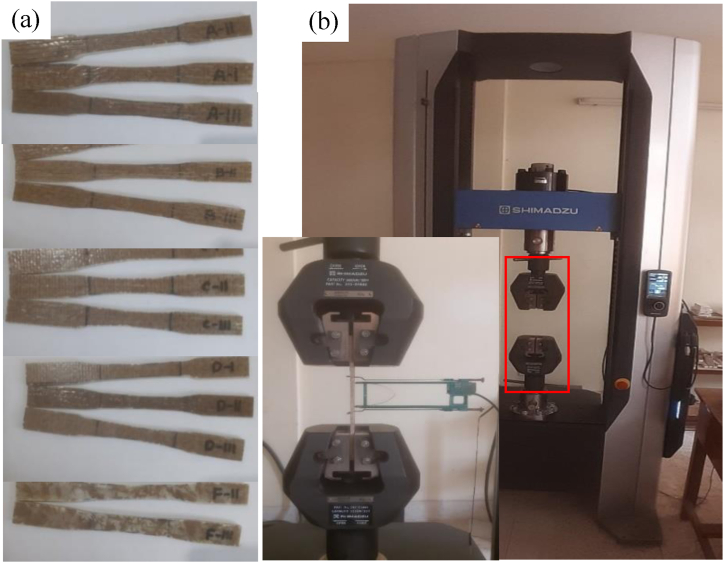
(a) Tensile test specimen photograph, from top Type A, B, C, D, and F, (b) Universal testing machine, inset: tensile test specimen with strain gauge.

The test was performed using a universal testing machine (UTM) supplied by Shimadzu AGS-X. For performing the tests, 2 mm/min crosshead speed and 50 mm gauge length were selected. The specimens were clamped on both ends in the clamps of the UTM. The specimens and the machine setup are shown in Fig. 6(a) and (b) respectively.

#### 2.4.2 Flexural properties

**Fig. 7:**
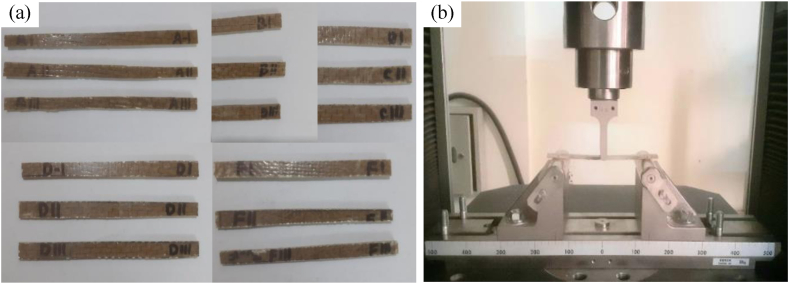
(a) Preparation of flexural samples, Type A, Type B, Type C, Type D, and Type, (b) Flexural test setup and specimen placed inside UTM.

The specimens were prepared according to the standard (ASTM D7264) for evaluating the flexural properties. The properties were found by performing three-point bending tests. According to the standard, the span to the thickness of the specimen was 32:1. Each specimen was 20 % longer than the support span length. The tests were performed in UTM with 3 mm/min crosshead velocity. The specimens and the setup for the test are shown in Fig. 7(a) and (b) respectively.

#### 2.4.3 Impact properties

**Fig. 8:**
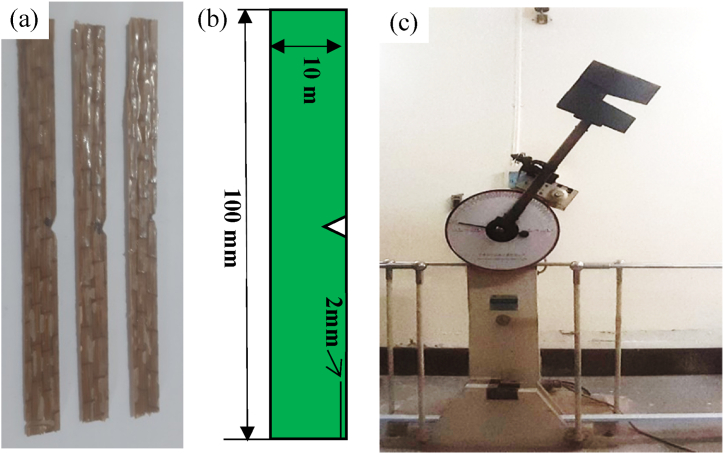
(a) Specimen prepared for impact test, (b) schematic diagram showing the dimensions of specimen, and (c) Charpy impact test machine.

A pendulum impact tester was used to conduct Charpy impact tests. According to the standard, the dimensions of each specimen were 100 mm × 10 mm, and a 2 mm deep 45° v-notch at the center of the specimen. The v-notch was introduced to localize the failure of the specimen. When a pendulum with a mass of 20 kg is dropped from the testing height of 143 cm, it has an impact on the composite sample. The tester computes and displays the impact energy absorbed by the samples and the specimen's impact strength. A Charpy Impact Testing machine carried out the impact experiments. The figures of the specimens prepared for impact tests, the dimension of the specimen, and the Charpy impact test machine are shown in Fig. 8(a)–. (b), and Fig. 8(c), respectively.

## 3 Results and discussion

As mentioned in Section 2, a large number of instruments and experimental setups were used in this study to analyze and characterize the properties of the fabricated composites. The results obtained from these are thoroughly discussed in this section. The fabricated composites' mechanical, physical, microstructural, and failure properties are examined.

### 3.1 Analysis of physical properties

#### 3.1.1 Density

**Fig. 9:**
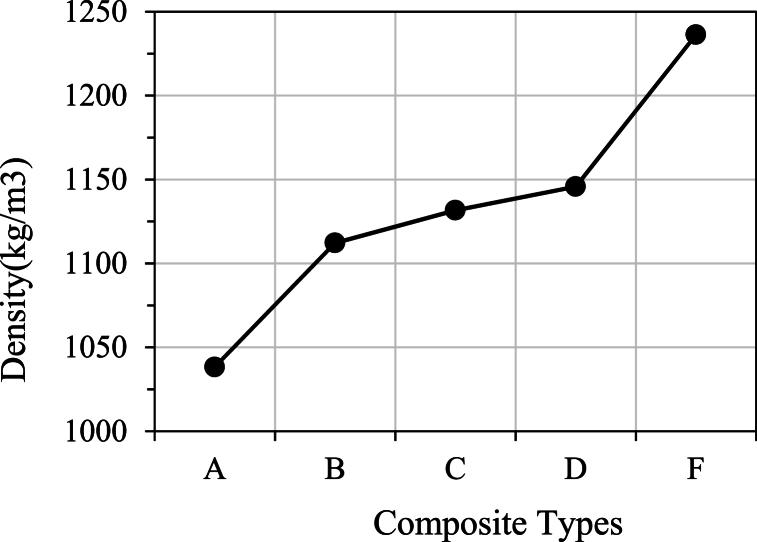
Determination of density.

Eqn. (1) is used to determine the density. Fig. 9 shows the result of the density measurement. 3 specimen were taken for each type of combination. As can be seen, Type A and Type F have the lowest and highest density, respectively. Density follows an upward trend depending on the number of glass layers used. Glass fiber is slightly heavier than jute fibers and contributes to higher density. Fig. 5 depicts that in Type A, only one layer of glass is used owing to minimum density. However, in Type B, C, and D, two glass layers are used, resulting in similar magnitudes of their density. In Type F, three layers of glass fibers are used, resulting in maximum density.

#### 3.1.2 Specific gravity

**Fig. 10:**
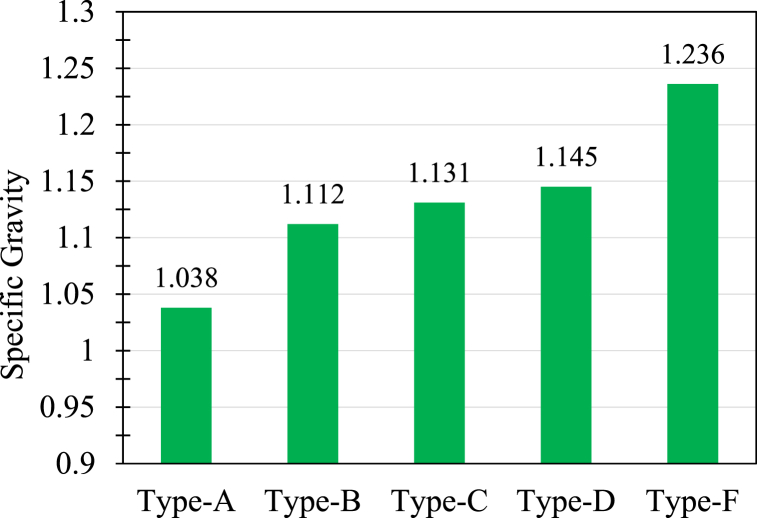
Specific gravity of the composites.

Specific gravity is the term that indicates how much an object is denser than water. It also indicates whether an object would float in water or not. The obtained specific gravity of the composites is presented in Fig. 10. For calculating specific gravity 3 samples were considered for each type of composite. It is clearly observed that Type A (J + J + G + J + J) has the lowest and Type F (G + J + G + J + G) has the highest specific gravity. The results are consistent with those obtained in density measurement. The variation of the magnitudes is related to the amount of heavier glass fiber employment. However, it is worth mentioning that all the values are more than unity because the composites are heavier than water of the same volume.

#### 3.1.3 Water absorption

**Fig. 11:**
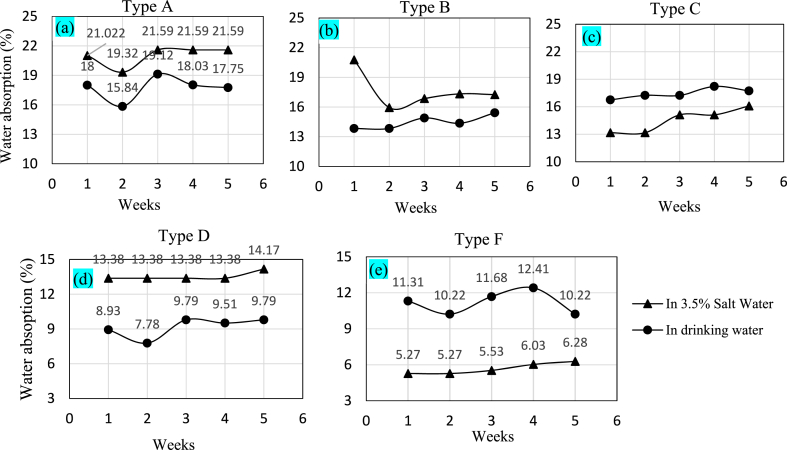
Water absorption of (a) Type A, (b) Type B, (c) Type C, (d) Type D, and (e) Type F.

The moisture content in jute-glass fiber composite significantly influences its durability and mechanical properties. A detailed discussion relating to water absorption of such composites can be found in Ref. [31]. The water absorption behavior of various orientations of hybrid composites was investigated during a weekly timeframe. The specimens were submerged in distilled and 3.5 % salt water to do so. After each week, the specimens were removed from the submerged condition, and all the surface water was wiped out before measuring the weekly weights of the specimens. The results are shown in Fig. 11. Six samples were prepared for each type of combination; three in drinking water and three in salt water.

It can be seen that the specimen of Type A (Fig. 11(a)) gained 18 % weight in the first week of immersion in normal drinking water and 21 % in salt water. This gain in weight is absorbed by water in the voids created inside the matrix and the gaps available inside the jute fibers. From the analysis of 5 weeks of data, it also can be seen that the water absorption rate is the highest during the first week of immersion; after that, it becomes nearly uniform in both types of water. This trend obtained is comparable to the study reported in Refs. [28,31]. Essentially, jute absorbed most of the water as it is built up of cellulose, which is a hydrophilic glucan polymer. The primary unit of jute fiber is anhydro-d-glucose, which consists three hydroxyl (–OH) groups [28]. In salt water, the specimen absorbed more because the salt created a corrosive environment for the natural jute fibers, which tend to delaminate the fibers from the matrix, causing more space to absorb saltwater and gain more weight. In type B (Fig. 11(b)), the specimen absorbed the highest percentage of water, 20.77 %, during the first week in salt water. There is sharp drop in the second week. This is because the both outer layers of Type B is jute leading to most of the water consumed in 1st week whereas the inner layers are glass fiber resulting in reduced consumption of water. After the second week and it remained almost the same until the 5th week. In normal drinking water, only 13.83 % was absorbed in the first week, and the trend of absorbing water remained almost constant. Compared with Type A, it absorbed less water as it contained less natural jute fiber layers.

The outer two layers in Type C are glass fibers (Fig. 5(c)). It absorbed a higher percentage of drinking water than salt water. Additionally, compared to Type A and Type B, Type C (Fig. 11(c)) absorbed less water because the natural fiber layers of this type were less exposed to water. The synthetic glass-fiber layers were fully exposed to water, which does not have excellent water absorption characteristics. This is the main reason why Type C absorbed a lesser percentage of water. Type D specimens absorbed only 8.93 % of water in the first week as shown in (Fig. 11(d)), which increased to 9.79 % in the fifth week. While immersed in salt water, the specimen absorbed 13.38 % of water in the first week and 14.17 % after 5 weeks. From this observation, we can say that the water absorption characteristic of the Type D hybrid is trending almost constantly, and its normal drinking water absorption capacity is less than that of the previous types. Among all the types, Type F showed the least salt water absorption as can be seen in (Fig. 11(e)). During the 5 weeks of data, its saltwater absorption varied from 5.27 % to 6.28 %. In addition, the specimen of Type F immersed in normal drinking water absorbed 11.31 % in the first week, and the absorption was reduced to 10.22 % after 5 weeks. This is only better than Type D. It is to be noted that water absorption, as examined in Ref. [28], is much higher than in our study, where they fabricated Jute fiber-reinforced polypropylene matrix composites. Thus, adding glass fibers helps to prevent water absorption. In such hybrid composites water absorption is important as it correlates to better wear resistance and durability [32].

#### 3.1.4 Swelling thickness test

**Fig. 12:**
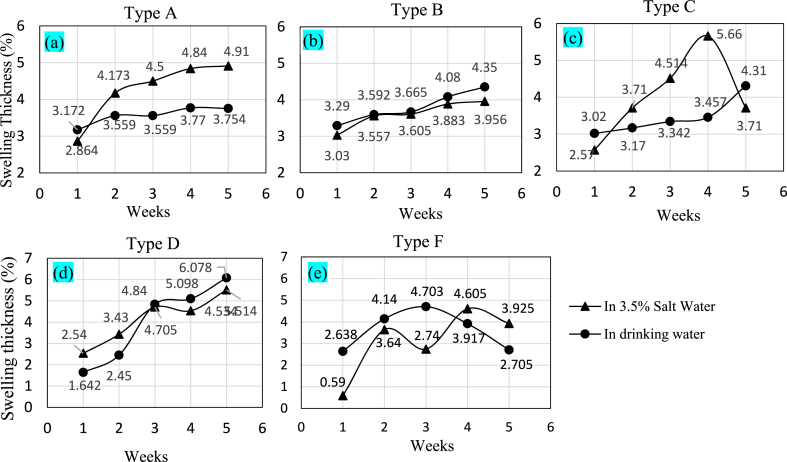
Swelling thickness of (a) Type A, (b) Type B, (c) Type C, (d) Type D, and (e) Type F.

This test was performed to determine if any swelling occurred during the immersion of the specimens while performing water absorption characteristics. For determining the swelling percentages of the specimens' thickness was recorded in weekly intervals and data from 5 weeks of observation is presented in Fig. 12. Similar to water absorption, six samples were prepared for each type of combination; three in drinking water and three in salt water.

As can be seen in Fig. 12(a), the thickness of the Type A specimen continuously swelled from 2.86 % to 4.91 % in the 5 weeks of observation time. However, the specimen immersed in normal water maintained swelling that was almost similar. Fig. 12(b) presents Type B, which shows almost the same swelling characteristic in both salt and normal drinking water. Like the previous type of composite, major swelling occurred after the first week of immersion. Then the swelling procedure slowed down for the next weeks of observations. For the specimen of Type C as shown in Fig. 12(c), swelling increased from 2.57 % to 5.66 % in 4-week intervals, but swelling decreased in salt water at the fifth week. So, the trend of swelling of Type C in salt water cannot be predicted from these data. Further observations are needed in this case. However, in normal drinking water, the swelling percentage increased weekly. Type D is the only composite in which the glass fiber layer and the jute layer had equal contact with water while being immersed (Fig. 12(d)). For immersion in both types of water, the specimen of Type D showed a similar trend of increasing swelling percentage. In normal water, swelling increased from 1.642 % to 6.078 %, and in saltwater, swelling changed from 2.54 % to 5.514 % in 5 weeks of observation. Fig. 12(e) shows specimen of Type F, where 3 layers of glass fibers and 2 layers of short jute fibers were sandwiched, showed the most unpredictable swelling characteristic. Due to the sandwiched short fibers, there were a lot of voids inside the matrix, and the adhesive could not be laid up evenly. During the 5 weeks of observations, swelling percentages increased and decreased in both saltwater and normal water. This is also worthy to note that, though there are many studies of swelling thickness of individual jute or glass fiber, or different combination of them with other fibers, a little is known about the swelling thickness of jute glass fiber reinforced epoxy composite. Thus, this paper gives an imperative information on the sweeling thickness of such composites.

### 3.2 Mechanical properties

#### 3.2.1 Tensile properties test

**Fig. 13:**
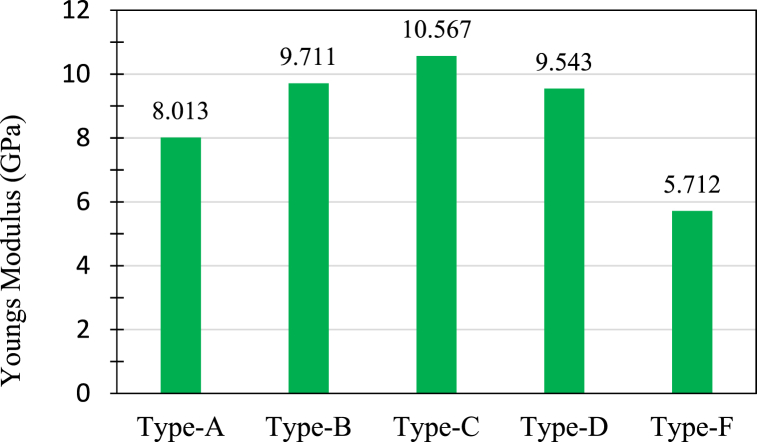
Young modulus of composites.

The tensile properties of the fabricated hybrid composites were determined using UTM. The obtained data is presented in Table 4. For each type 3 specimen were prepared and named such as A1, A2, and A3. It can be seen that Type A (J + J + G + J + J) could bear the maximum tensile load among all the hybrids as it has the maximum thickness. Young's modulus of the specimens was determined from the most linear part of the stress-strain curve. The variation of Young's modulus due to stacking orientation can be seen in Fig. 13.

**Table 4:** Results of the tensile tests done on the composite specimens.

Type of Composite	Specimen	Strength (MPa)	Average strength (MPa)	Peak Load (KN)	Average Peak Load (KN)	Youngs Modulus (GPa)	Ave. Youngs Modulus (Gpa)
A	A1	91.9192	93.996	9.00073	9.159	8.0032	8.013
	A2	91.0106		9.11162		7.6662	
	A3	99.0583		9.36596		8.3717	
B	B1	122.905	121.134	8.06258	7.898	10.172	9.711
	B2	119.364		7.73475		9.2519	
C	C1	105.915	115.284	6.11659	6.532	11.300	10.567
	C2	118.512		6.67817		10.096	
	C3	121.426		6.79987		10.307	
D	D1	104.706	109.904	6.19439	6.628	9.9972	9.543
	D2	133.595		7.73996		9.6273	
	D3	115.102		7.06148		9.0064	
F	F1	64.6464	63.938	6.31272	6.264	5.0511	5.712
	F2	57.9943		5.30648		6.5547	
	F3	69.1727		7.1732		5.5313

It can be seen that Type C(G + J + J + G) shows the best tensile properties among all the laminates. Among the hybrids, Type A and Type F have 5 layers of fibers, whereas the others have 4 layers. But from the test results, it is clear that the 4 layers composites have shown better tensile strength. The reason can be identified as the lack of enough glass-fiber layers in Type A and the absence of continuous jute fiber layers in Type F. Stacking layers play a vital role in determining the mechanical properties of jute-fiber composites, as reported in Ref. [33].

**Fig. 14:**
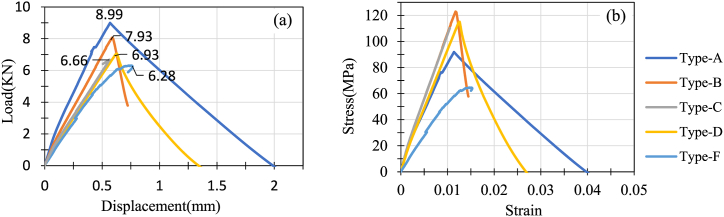
(a) Load vs displacement curve for tensile load, (b) stress vs strain curve for tensile load.

The load vs displacement curve (Fig. 14((a)) shows that Type A could take more load than any other composites for a definite displacement. Type F displaced more than other composites prior to tensile loads because of 3 layers of glass fiber. Type B, which has 2 layers of glass fibers as the center core, showed the second-best load-taking capacity for a definite displacement. Ruhul et al. [28] fabricated jute fiber/pp composite and E-glass fiber/pp composite and reported a maximum tensile strength and modulus of 85 MPa and 7 GPa. Likewise, Raman et al. [34] studied jute/carbon fibre reinforced epoxy composite and reported 13.37 MPa tensile strength for jute epoxy hybrid composite. Therefore, the results presented in this study are significantly higher than previous studies. The improvement of tensile properties is attributed to addition of glass fibers. This is also aligned with the findings reported by Sanjay et al. in Ref. [35] and Vijay et al. in Ref. [36].

**Fig. 15:**
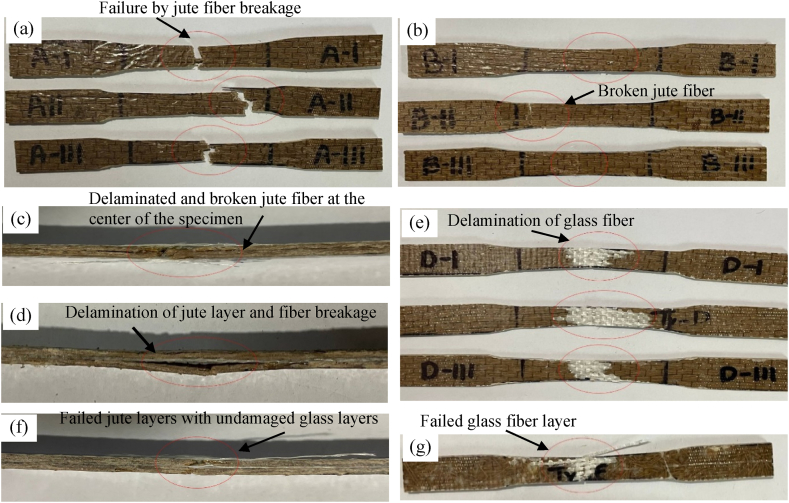
Representative featured photographs of failed specimen (a) Type A top view, (b) Type B top view (c) Type B side view, (d) Type C side view, (e) Type D top view, (f) Type D side view, and (g) Type F top view.

Fig. 14(b) shows stress-strain curves plotted above for all the hybrids. It is observed that Type B(J + G + G + J) and Type C(G + J + J + G) followed almost the same stress vs strain properties. But Type B has more yield strength than Type C, so it could take more tensile stress. Even with a lower Young's modulus Type D could withstand more tensile stress than Type C. Due to less glass fibers layer, Type A could take less tensile stress than Type B, Type C, and Type D. On the other hand, Type F was fabricated with short jute fibers which were randomly distributed and sandwiched between 3 glass-fiber layers. As there is no continuous jute yarn, its specimens could not distribute the stress properly, so Type F showed the lowest tensile property and failed at the lowest stress. The representative featured failed pictures of the specimens shown in Fig. 15.

Due to tensile load, the specimens of Type A showed similar types of failure (Fig. 15(a)). All 3 specimens suffered jute fiber breakage. Only one 18GSM glass fiber layer was not enough to hold the two sides of the specimens; as a result, all the specimens broke into two separate parts. As discussed earlier, Type B showed the best tensile stress vs strain properties. Fig. 15(b) and (c) presents the top and side view of delamination and breakage of jute fiber of specimen Type B respectively. From the analysis of the images, it is noticed that the failure is caused by fiber breakage. At first, the matrix between the jute layers and glass fiber layers of Type B failed. Then the specimen continued to take a tensile load until the jute fibers broke into two parts. In the case of Type C, two glass fiber layers are laminated over two center jute layers. Due to tensile loading, the matrix between the outer glass layers and center jute layers failed, and then the glass fiber layers were detached from the jute. Then, the center jute part broke into two parts, and the specimen failed. Fig. 15(d) shows the side view of delaminated and broken jute fiber at the center of specimen Type C. Type D combines glass and jute fibers on its outermost layers. During the tensile test, the matrix between glass and jute failed, and the glass layer was delaminated from the jute layer next to it. Fig. 15(e) shows the top view of delamination of glass fiber of specimen Type D, and Fig. 15(f) presents the side view of failed jute fiber with undamaged glass fiber of specimen Type D. After delamination of the glass layers from the adjacent jute layers, the jute layers were not strong enough to take more stress, and the specimen failed as the advantage of having 3 glass fiber layers. Type F displaced or elongated more than other types due to tensile loads. However, as a consequence of the lack of continuous jute, it failed to compete with other types of tensile stress bearing. The failure of the specimens of Type F mainly occurred by the glass fiber layer's breakage following the glass layers' delamination from the short jute fibers as shown in the top view of failed glass fiber in Fig. 15(g). The following figure shows that the randomly distributed short fibers did not help the composite take large tensile stress.

#### 3.2.2 Flexural strength test

**Fig. 16:**
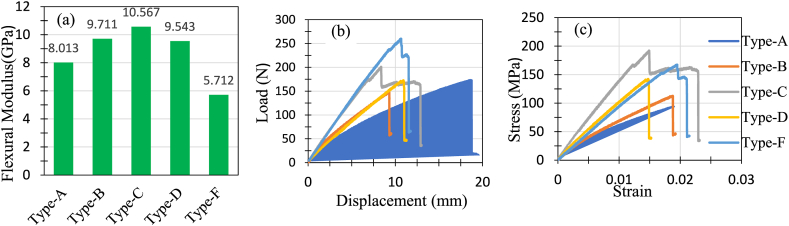
(a) Flexural modulus for the fabricated composites, (b) load vs displacement curve for flexural load on the composites, (c) stress vs strain curve for flexural load.

A 3-point bending test in UTM determined the flexural properties of the 5 different types of hybrid composites. The flexural test results are presented in Table 5. Similar to tensile test, 3 specimen were prepared for each type of combination and named them such as B1, B2, and B3The table showed that Type C(G + J + J + G) exhibited the best result for flexural strength with 217.467 MPa and flexural Young's modulus with 13.872 GPa. Raman et al. [34] reported flexural strength and Young's modulus of jute epoxy hybrid composite of 104 Mpa and 1.225 GPa respectively. Thus the addition of glass fibers significantly improved the flexural properties. Such attributes are also in alignment with the results reported in Ref. [7]. Meanwhile, Type F(G + J + G + J + G) required a maximum flexural load of 287.288 N before failure took place. Each type's flexural modulus for flexural loads was determined from the most linear part of the stress-strain curve of each type's specimen. Then, average values were taken, and the results are shown in Fig. 16(a).

**Table 5:** Results from the flexural properties tests.

Type of Composite	Specimen	Flexural Strength (MPa)	Average flexural strength (MPa)	Peak Load (N)	Avg. Peak Load (N)	Flexural Modulus (Gpa)	Avg. Flexural Modulus (Gpa)
A	A1	113.77	101.053	203.016	176.490	4.9906	4.824
	A2	86.7784		153.432		5.0826	
	A3	102.61		173.022		4.3990	
B	B1	98.1285	109.459	119.175	146.974	7.4973	7.913
	B2	117.511		173.592		8.1911	
	B3	112.738		148.155		8.0533	
C	C1	192.241	217.467	201.063	235.332	13.483	13.872
	C2	268.087		294.894		13.145	
	C3	192.073		210.039		14.988	
D	D1	142.382	147.681	172.605	176.386	9.6516	9.561
	D2	140.706		177.903		9.2901	
	D3	159.957		178.65		9.7407	
F	F1	177.518	158.088	346.026	287.288	9.2877	8.966
	F2	167.539		260.082		9.0678	
	F3	129.208		255.756		8.5419

**Fig. 17:**
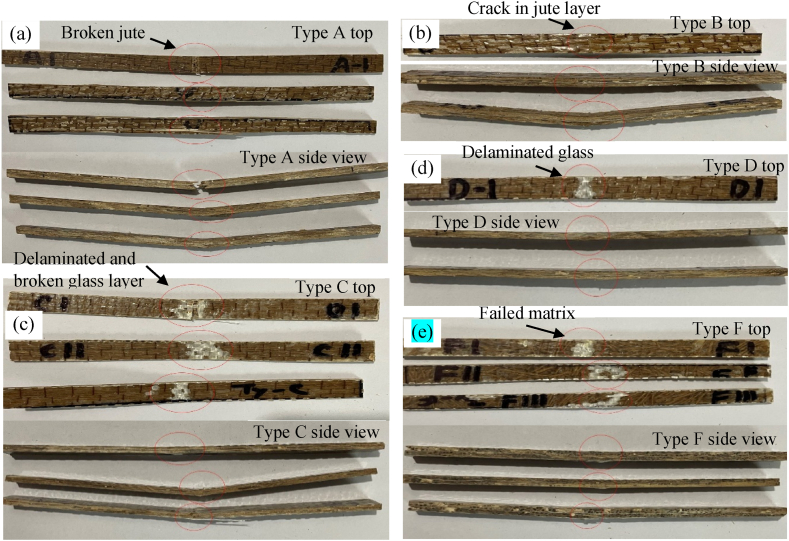
Top and side views of fractured specimens (a) Type A, (b) Type B (c) Type C, (d) Type D (e) Type F.

Fig. 16(b) presents the load and displacement curve under flexural load. The figure shows that the Type A hybrid possessed maximum strain, whereas Type F took maximum load before failing. Type C composite was the only type that had strain before failing nonlinearly, even after the yield force. Meanwhile, the other types of composites failed immediately after the yield force. From the stress-strain curves of the composites (Fig. 16(c)), it is clear that Type C(G + J + J + G) showed the best stress vs strain properties. At the same time, Type D (J + G + J + G) failed with the lowest strain applied. In the case of Type C, the outer two glass fiber layers are the main factors responsible for its best flexural properties. The specimens after failure due to flexural loading are depicted in Fig. 17.

From the above-fractured pictures of Type A, as shown in Fig. 17(a), it is clear that due to 3-point bending, the upper surfaces of the specimen faced contraction, and the lower surfaces faced expansion. When the downward forces reached the capacities of the specimens, they failed due to fiber breakage. The fractured specimens of Type B (Fig. 17(b)) show that all the specimens failed due to fiber breakage of the jute mat. It can be seen that the glass-fiber layer didn't fail at all in the case of Type B, as the upper surface and the lower surface are made of jute. Poor contraction and expansion properties of jute compared to glass fiber layers are the main reasons Type B composites fail so early due to flexural load. In Type C (Fig. 17(c)) composition, the glass fiber layers make the upper and lower surfaces. So, the glass fiber layers took the maximum amount of flexural loading, making Type C exhibit the best property under flexural loading. From the fractured specimens, it is evident that failure was initiated due to the matrix failure. In Type D (Fig. 17(d)), the glass fiber layer is on the upper surface, and the jute is at the bottom. As a result, due to flexural load, only the jute layer at the lower surface failed. The glass fiber layer at the top only got delaminated and slightly detached from the adjacent jute. Fig. 17(e) shows the failure criteria of Type F under flexural load. The load-displacement curve shows that the Type F hybrid failed after taking the maximum load among other types. Analyzing the failure criteria can be described as the result of 3 layers of glass fiber, which gave Type F the most flexural load-carrying capacity. However, the loads could not be distributed evenly all over the specimen due to the absence of continuous jute. This is the reason Type F could not show satisfactory stress characteristics against strain. Similar studies demonstrated a similar outcome [37,38].

#### 3.2.3 Impact properties test

**Fig. 18:**
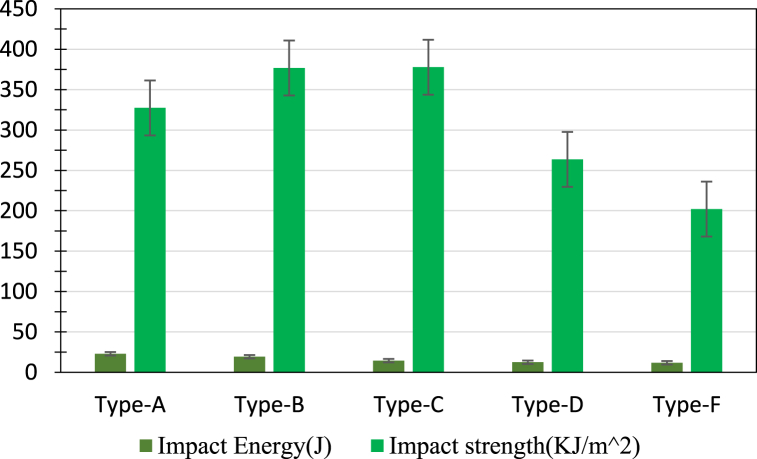
Impact energy and impact strength of the fabricated composites.

**Fig. 19:**
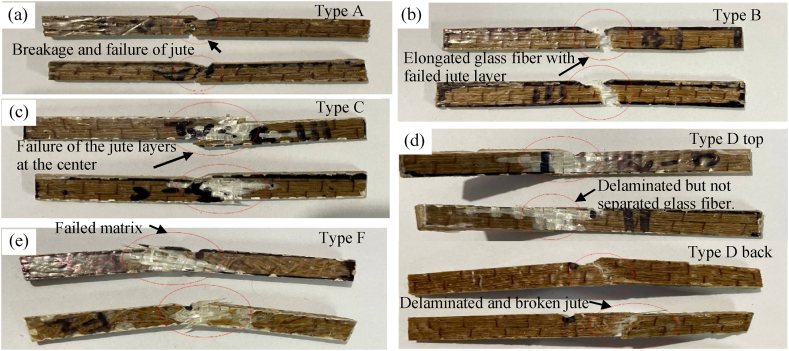
Fracture criteria under impact load of all types of hybrid composite materials (a) Type A, (b) Type B, (c) Type C, (d) Type D – top and bottom surface, (e) Type F.

Charpy impact tests were performed to evaluate the impact strength properties of the hybrid composites. Fig. 18 shows the comparison of impact strength and energy absorbed by the composites. Three samples were prepared for each type of composite. It can be seen that Type A (J + J + G + J + J) has absorbed the most energy among all the other composites due to its thickness. However, in terms of impact strength, Type C (G + J + J + G) is stronger than other types of composites. It can also be noted that Type B (J + G + G + J) has almost a similar impact strength to Type C, which is far more than the remaining ones. Two glass fiber layers at the outermost parts of Type C and two glass fiber layers at the center of Type B contributed to their better impact resistive strength. Impact strength obtained is considerably better than previous results as reported in Ref. [38], where authors used hybrid composites jute fiber and S-glass fibers with epoxy resin. It can be understood more clearly by analyzing the fracture criteria of the specimens after the impacts of the hammer of the Charpy test. Fig. 19 illustrates the fracture criteria of all types of hybrid composites.

In Type A (Fig. 19(a)), 4 layers of jute and 1 layer of 18 GSM glass fiber are at the center. Due to the lack of enough glass fiber layers that can elongate more than the jute fibers, all the specimens of Type A failed completely and couldn't show satisfactory impact strength relative to their thickness. In Type B, even after the failure of the outermost jute layers due to high impact, the two glass fiber layers at the center didn't completely fail. Instead, it elongated and increased the impact strength of the specimens of Type B, as mentioned in Fig. 19(b). In the case of Type C (Fig. 19(c)), the two glass fiber layers are the outermost layers that absorb the most impact energy and prevent the central jute layers from failing so easily. As a result, the Type C hybrid showed the best impact strength property. During the impact tests on the specimens of Type D, the glass layer was put under the impact load. Due to the high impact load of the hammer, the glass fiber layer gets delaminated from the jute layer next to it (Fig. 19(d)). This delamination procedure propagates to the last jute layer, and as a consequence, the previous jute layer failed completely, and Type D showed not much satisfactory impact strength compared to Type B and Type C. Type F has 5 layers of fibers, where short jute fibers were randomly distributed and sandwiched between 3 glass fiber layers. So, the glass layer took the impact load for all the specimens. But from the figure, the failures didn't occur at the v-notch but rather at the places where jute and matrix were less relative to adjacent areas (Fig. 19(e)). Along with that, due to the absence of continuous jute fibers, the impact load couldn't be distributed along the fiber length, so Type F specimens failed so easily and possessed the lowest impact strength property among all the fabricated hybrids.

Determining the physical and mechanical properties of the hybrid composite play a significant role in applying them in practical applications. For example, Fitri et al. [39] investigated the mechanical properties of the interior component (dashboard and interior wall) of automotive vehicles. The authors reported the range of various mechanical properties for such applications including tensile strength from 20 to 40 MPa, Modulus of elasticity in between 1 and 2.5 Gpa (1000–2500) MPa, and impact strength of about 13.48 kJ/m^2^. Similare observations were also preported in Ref. [40]. The mechanical properties of composites fabricated in this study shows better results compared to aforementioned, and thus perfectly suitable for the mentioned applications. Sujan et al. [41] extensively reported the applications of natural fiber based composites in furniture, construction, packaging, and shipping pallets. The physical and mechanical properties required for such applications are obtained in the hybrid composite presented in this study. Therefore, it is apparent that the composite has ample potential applications in various sectors to replace wood, plastic, or metallic materials for lightweight and better durability. An in-depth review of industrial applications of such composites can also be found in Ref. [27].

### 3.3 Microstructure analysis

**Fig. 20:**
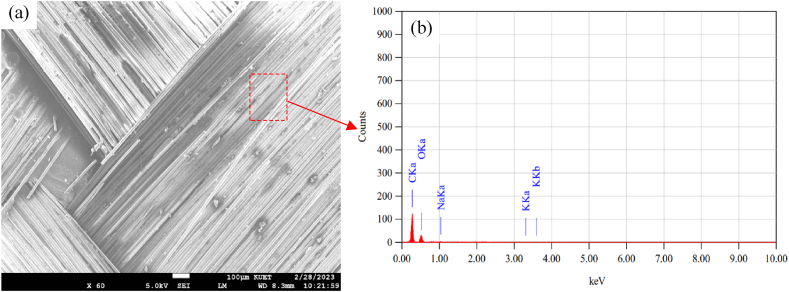
(a) SEM micrograph, (b) EDS analysis of the Jute fibers.

**Fig. 21:**
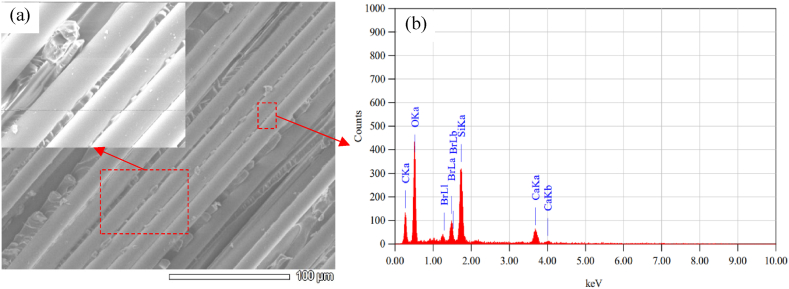
(a) SEM micrograph, (b) EDS analysis of the composites of the Glass fiber.

The micrograph of base materials and the fracture analysis have been carried out extensively by a scanning electron microscope (SEM) to observe the microscopic materials' properties that influence the macroscopic behavior of the composites. Also, the chemical composition of the glass fibers and the jute fibers were analyzed using Energy Dispersive Spectroscopy (EDS). A representative areas of the SEM data were selected to analyze for EDS measurement. Are is mentioned using dashed red color box. Fig. 20(a) and (b) show representative SEM micrograph and the results of EDS analysis of jute fiber. Correspondingly, Fig. 21(a) and (b) show representative SEM micrograph and the results of EDS analysis of glass fiber. The results show that the Jute fibers contain mainly carbon and oxygen, whereas the glass fiber consists of Silicon, Oxygen, and Carbon.

**Fig. 22:**
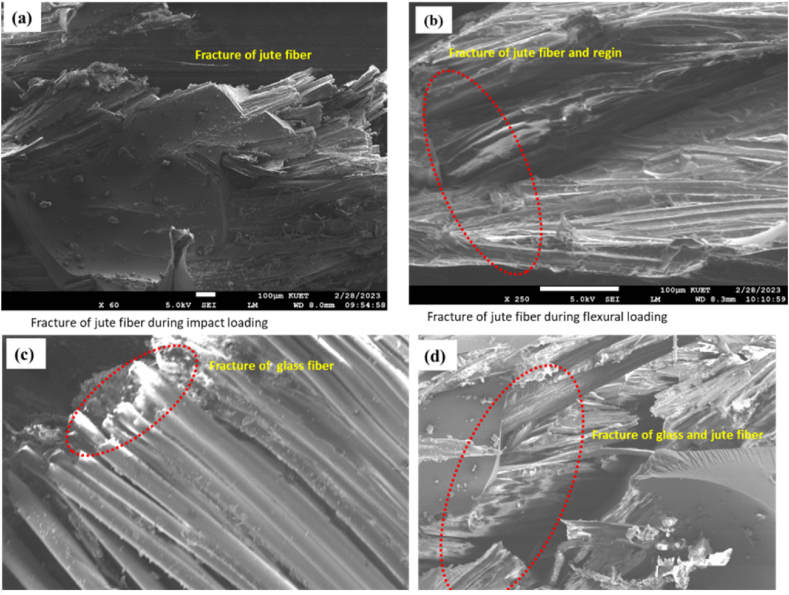
Representative SEM images of fractured surfaces: (a) jute fiber due to impact loading, (b) jute fiber due to flexural loading, (c) glass fibers due to impact loading, and (d) glass and jute fiber due to impact loading.

The representative failure mode of the composites was investigated from the SEM data of the flexural test samples. Fig. 22 shows the SEM images of the fracture surface of the fibers. It is observed that the occurrence of jute fibers and glass fibers is predominantly due to fiber breakage. Significant incisions were also noted on the jute fibers, as is shown in Fig. 22(a) and (b). Their fibrous composition indicates the presence of brittleness in jute fibers. The results are also comparable to those of the study performed in Ref. [29]. It was noticed during the testing that matrix cracking typically happens prior to structural breakdown when flexural stress is imposed. The observation of jute fiber breakage in Fig. 22(a) and (d) and the stretching of glass fiber from the matrix in Fig. 22(c) are evident in the analysis of the testing specimen.

SEM micrographs also show small gaps in the matrix near the jute fibers. This indicates that the jute fiber pull-out is relatively higher, and the bonding between jute and glass fibers is not so good. It also reported very few holes in the matrix, suggesting very good bonding between the glass fiber and the matrix. The testing results of Jute and glass fiber-dominated composites also show that jute-based composites have comparatively low mechanical and interfacial properties compared to glass-based composites. In general, the interfacial connection between the matrix phase and fibers exhibits significant strength up to the point of elastic limit. Nevertheless, once the elastic limit is surpassed, the adhesion properties exhibit a significant decline in strength. Therefore, a further investigation will have to be carried out to reduce the hydrophilic nature of jute and to try to improve the interfacial bonds with jute fiber, retaining the inherent biodegradable properties of jute fibers. This research opens new doors for further study to bring the mechanical properties of jute composites closer to that of the glass-based composite.

## 4 Conclusion

In this experiment, a novel jute-glass fiber hybrid composite was successfully fabricated. Five distinct layer combinations were evaluated to identify the optimal configuration. A variety of tests were conducted using different instruments and experimental setups to thoroughly characterize the physical, mechanical, failure, and microstructural properties of the composites. The results obtained were extensively discussed, highlighting both the potential and limitations of the materials. When compared to previous studies, significant improvements were observed, suggesting strong possibilities for commercial applications.

Key findings include.

• **Impact Energy Absorption**: Type A (J + J + G + J + J) absorbed the most impact energy, while Type C (G + J + J + G) exhibited the highest impact strength.
• **Tensile Performance**: Type A (J + J + G + J + J) supported the maximum load, whereas Type E (G + J + G + J + G) showed the greatest displacement under tensile loading. Type B (J + G + G + J) recorded the highest tensile strength, and Type C (G + J + J + G) achieved the maximum Young's modulus.
• **Flexural Properties**: In flexural tests, Type E (G + J + G + J + G) withstood the highest load before failure, while Type A (J + J + G + J + J) exhibited the most deflection prior to fiber cracking. Additionally, Type C (G + J + J + G) demonstrated the highest flexural strength and modulus.

Combining results from impact, tensile, and flexural tests indicates that Type C (G + J + J + G) outperformed the other composites. Physical property tests on water absorption and swelling revealed that Type F (G + J + G + J + G) exhibited the lowest absorption and swelling rates. This suggests that the incorporation of glass fibers in the outer layers, along with the use of woven jute fibers, enhanced the mechanical properties while reducing water absorption and swelling.

However, it is important to note that the inclusion of short jute fibers negatively impacted the mechanical properties. EDS analysis and SEM micrographs provided insights into the nature of the Jute-Glass Fiber Reinforced Epoxy Composites under mechanical loading. The fiber material demonstrated semi-brittle characteristics, with increased strength observed in the outer glass fiber layers. Typically, the fibers displayed greater strength than the matrix, leading to matrix cracking occurring before fiber failure. Overall, this study underscores the potential of jute-glass fiber composites for various applications, emphasizing their mechanical advantages and durability.

## CRediT authorship contribution statement

**Md Mahadi Hasan:** Writing – review & editing, Writing – original draft, Methodology, Data curation, Conceptualization. **Md Ashraful Islam:** Writing – review & editing, Writing – original draft, Visualization, Investigation, Formal analysis, Data curation, Conceptualization. **Tareq Hassan:** Writing – review & editing, Writing – original draft, Validation, Methodology, Formal analysis, Conceptualization.

## Data and code availability statement

Data is available on request. No code was used for the research described in the article.

## Funding statement

This research did not receive any specific grant from funding agencies in the public, commercial, or not-for-profit sectors.

## Declaration of competing interest

The authors declare that they have no known competing financial interests or personal relationships that could have appeared to influence the work reported in this paper.

## References

[1] Hasan M., Zhao J., Jiang Z. (2019). Micromanufacturing of composite materials: a review. Int. J. Extrem. Manuf..

[2] Park S.-J., Seo M.-K., Park S.-J., Seo M.-K. (2011). Interface Science and Technology.

[3] Yashas Gowda T.G. (2018). Polymer matrix-natural fiber composites: an overview. Cogent Engineering.

[4] Keya K.N. (2019). Natural fiber reinforced polymer composites: history, types, advantages and applications.

[5] Thyavihalli Girijappa Y.G. (2019).

[6] Townsend T. (2020). Handbook of Natural Fibres.

[7] Jawaid M., Khalil H.A.J.C.p. (2011). Cellulosic/synthetic fibre reinforced polymer hybrid composites: A review.

[8] Pickering K. (2008).

[9] Thomas.Net, *Types Of Resins And Their Uses*. Thomas-a xometry company.

[10] Fastfix (2020). Knowledge of Material.

[11] Rahman A. (2023). Fabrication and performance investigation of natural-glass fiber hybrid laminated composites at different stacking orientations. J. Nat. Fibers.

[12] Rao D.N. (2020).

[13] Saba N., Md Tahir P., Jawaid M.J.P. (2014). A review on potentiality of nano filler/natural fiber filled polymer hybrid composites.

[14] Hasan M. (2020). Microstructural evaluation of WC and steel dissimilar bilayered composite obtained by spark plasma sintering. Int. J. Adv. Des. Manuf. Technol..

[15] Yadav P. (2022).

[16] Jani S. (2021).

[17] Song X. (2022). The reinforcement and toughening of jute/PLA (lactic acid) composite material with modified-rubber/SiO2 core-shell particles. Materials Today Sustainability.

[18] Rahman A. (2023). Fabrication and performance investigation of natural-glass fiber hybrid laminated composites at different stacking orientations.

[19] Turna, R.N., et al., Effect of Length of Composite on CFRP Strengthened Steel Beam-Column Joints under Cyclic Load.

[20] Mishra S. (2003). Studies on mechanical performance of biofibre/glass reinforced polyester hybrid composites.

[21] Gopinath A., Kumar M.S., Elayaperumal A.J.P.E. (2014).

[22] Prasad L., Saini A., Kumar V. (2021). Mechanical performance of jute and basalt fiber geo-grid-reinforced epoxy hybrid composite material. J. Nat. Fibers.

[23] Premnath A.A. (2019). Impact of surface treatment on the mechanical properties of sisal and jute reinforced with epoxy resin natural fiber hybrid composites. J. Nat. Fibers.

[24] Seldon P.A., Rajesh R. (2022). Mechanical and thermal characterization of hemp/rice-husk/E-glass fiber cardanol epoxy matrix hybrid composites. J. Nat. Fibers.

[25] Abd El-Baky M.A. (2022). Fabrication of cost effective fiber metal laminates based on jute and glass fabrics for enhanced mechanical properties. J. Nat. Fibers.

[26] Satapathy A. (2010). Processing and characterization of jute-epoxy composites reinforced with SiC derived from rice husk.

[27] Sanjay M.a. (2016). Studies on mechanical properties of jute/E-glass fiber reinforced epoxy hybrid composites.

[28] Khan R.A. (2010). Comparative studies of mechanical and interfacial properties between jute and E-glass fiber-reinforced polypropylene composites.

[29] Srivastav A. (2007). Loading rate sensitivity of jute/glass hybrid reinforced epoxy composites: effect of surface modifications.

[30] Chaudhary V., Bajpai P.K., Maheshwari S. (2018). Studies on mechanical and morphological characterization of developed jute/hemp/flax reinforced hybrid composites for structural applications. J. Nat. Fibers.

[31] Aquino E.M.F. (2007). Moisture effect on degradation of jute/glass hybrid composites. J. Reinforc. Plast. Compos..

[32] Abd El–Baky M.A., Kamel M. (2021). Abrasive wear performance of jute–glass–carbon-reinforced composites: effect of stacking sequence and fibers relative amounts. J. Nat. Fibers.

[33] Nunna S. (2012). A review on mechanical behavior of natural fiber based hybrid composites.

[34] Ramana M.V., Ramprasad S.J.M.T.P. (2017). Experimental investigation on jute/carbon fibre reinforced epoxy based hybrid composites.

[35] Sanjay M.R., Arpitha G., Yogesha B.J.M.t.p. (2015). Study on mechanical properties of natural-glass fibre reinforced polymer hybrid composites: a review.

[36] Chaudhary V., Bajpai P.K., Maheshwari S. (2018). Studies on mechanical and morphological characterization of developed jute/hemp/flax reinforced hybrid composites for structural applications. J.Nat. Fibers.

[37] Braga R.A., Magalhaes P.A.A. (2015). Analysis of the mechanical and thermal properties of jute and glass fiber as reinforcement epoxy hybrid composites. Mater. Sci. Eng. C.

[38] Sriranga B.K., Kirthan L.J., G A. (2021). The mechanical properties of hybrid laminates composites on epoxy resin with natural jute fiber and S-glass fibers. Mater. Today: Proc..

[39] Fitri M. (2020). The mechanical properties requirement for polymer composite automotive parts—a review.

[40] Herwandi, N R. (2017). Improved quality of recycled fiber for composite materials as motorized vehicle materials. Machine. J. Mech. Eng..

[41] Sujon M.A.S., Habib M.A., Abedin M.Z. (2020). Experimental investigation of the mechanical and water absorption properties on fiber stacking sequence and orientation of jute/carbon epoxy hybrid composites. J. Mater. Res. Technol..

